# Comparative Transcriptomic and Proteomic Analyses Provide New Insights into the Tolerance to Cyclic Dehydration in a Lichen Phycobiont

**DOI:** 10.1007/s00248-023-02213-x

**Published:** 2023-04-11

**Authors:** Eva M. del Campo, Francisco Gasulla, Aline F. Hell, María González-Hourcade, Leonardo M. Casano

**Affiliations:** 1 Department of Life Sciences, University of Alcalá, 28805 Alcalá de Henares (Madrid), Spain; 2 Centre of Natural Sciences and Humanities, Federal University of ABC, 09606-070 São Bernardo Do Campo, SP Brazil; 3 Department of Forest Biomaterials and Technology, Swedish University of Agricultural Sciences, 90183 Umeå, Sweden

**Keywords:** Dehydration, Rehydration, Microalgae, *Coccomyxa simplex*, Proteome, Transcriptome

## Abstract

Desiccation tolerance (DT) is relatively frequent in non-vascular plants and green algae. However, it is poorly understood how successive dehydration/rehydration (D/R) cycles shape their transcriptomes and proteomes. Here, we report a comprehensive analysis of adjustments on both transcript and protein profiles in response to successive D/R cycles in *Coccomyxa simplex* (Csol), isolated from the lichen *Solorina saccata*. A total of 1833 transcripts and 2332 proteins were differentially abundant as a consequence of D/R; however, only 315 of these transcripts/proteins showed similar trends. Variations in both transcriptomes and proteomes along D/R cycles together with functional analyses revealed an extensive decrease in transcript and protein levels during dehydration, most of them involved in gene expression, metabolism, substance transport, signalling and folding catalysis, among other cellular functions. At the same time, a series of protective transcripts/proteins, such as those related to antioxidant defence, polyol metabolism and autophagy, was upregulated during dehydration. Overall, our results show a transient decrease in most cellular functions as a result of drying and a gradual reactivation of specific cell processes to accommodate the hydration status along successive D/R cycles. This study provides new insights into key mechanisms involved in the DT of Csol and probably other dehydration-tolerant microalgae. In addition, functionally characterising the high number of genes/proteins of unknown functions found in this study may lead to the discovery of new DT mechanisms.

**Supplementary Information:**

The online version contains supplementary material available at 10.1007/s00248-023-02213-x.

## Introduction

Desiccation tolerance (DT), defined as a set of strategies that permit survival with a cellular water content equal to or less than 10% of dry weight and a water potential of − 100 MPa and below, is present in diverse organisms across all kingdoms of life including both prokaryotes and eukaryotes [1-3]. Photosynthetic organisms that are tolerant to desiccation have been widely investigated to improve drought tolerance in crop plants. Some angiosperms (resurrection plants), pteridophytes, bryophytes, lichens, aerial microalgae and terrestrial cyanobacteria are included among these photosynthetic organisms. DT is well-documented for lichen-forming microalgae [4]. However, little is known about the mechanisms of DT in these organisms compared to other photosynthetic eukaryotes.

Lichens are symbiotic associations of a fungal mycobiont, one or more green algal and/or cyanobacterial photobionts and many other microorganisms, such as bacteria, additional algae and fungi. Lichens are extraordinarily resistant to adverse environmental conditions in a desiccated status [5]. Thus, DT can be regarded as an adaptive strategy for surviving in stressful environments that allows lichens to conquer diverse geographical areas where most organisms do not thrive. Both photobionts and mycobionts appear to be dehydration tolerant and recover within minutes of rehydration. However, the DT is species-specific and depends on the speed, intensity and duration of water loss during dehydration and environmental conditions [6]. Most chlorobionts are classified as the Trebouxiophyceae class, which also include free-living terrestrial microalgae. More than half of Lecanoromycetidae lichen species associate with microalgal species of the genera *Trebouxia* Puymaly or *Asterochloris* Tschermak-Woess. Both algal genera include desiccation-tolerant species/strains and dominate in lichens that inhabit ecosystems where the climate prevents most living beings from surviving [7].

The trebouxiophyte genus *Coccomyxa* Schmidle includes species distributed worldwide, which can be found free living and in symbiotic associations colonising terrestrial and aquatic habitats. *Coccomyxa* microalgae associate with a variety of partners, including fungi (asco- and basidiomycetes) in obligate or facultative symbiosis, vascular plants and aquatic invertebrates in mutualistic or parasitic associations [8]. *Coccomyxa simplex* (Csol) and *Trebouxia* sp. (TR9) are two lichen microalgae with contrasting strategies to cope with dehydration/rehydration (D/R), which are related to the ecological context in which each microalga thrives. TR9, one of the phycobionts of the lichen *Ramalina farinacea*, is well adapted to environments with rapid diurnal and/or seasonal D/R cycles, whereas Csol, the phycobiont of the lichen *Solorina saccata*, is well adapted to protected mild and cold environments with less drastic dehydration conditions [9].

Desiccation-tolerant organisms have to meet the challenges of dehydration to prevent cellular damage during D/R [10]. The mechanisms underlying DT have been understudied in lichen-forming green algae (reviewed in [4]). Dehydration affects photosynthesis, which is usually completely blocked. Differential DT across green algal species, assessed from photosynthetic efficiency during D/R cycles, was accompanied by differential accumulation of intracellular reactive oxygen species. Dehydration also affects the structural integrity of the cell, especially its membranes and cell wall. Desiccation-tolerant cells protect their membranes and cell wall from water loss during dehydration by increasing their fluidity and flexibility. Although most of these protective mechanisms are constitutive, some of them are inducible [4].

It is currently difficult to investigate the mechanisms underlying DT at the molecular level in green microalgae, particularly in lichen-forming chlorophytes, which is mainly due to the scarcity of available genomic and/or transcriptomic and/or proteomic information. The transcriptomic approach applied to *Trebouxia gelatinosa*, desiccation-tolerant lichen-forming green microalgae after a single D/R cycle, supported the notion that DT is primarily based on constitutive mechanisms [11] as in other previously studied lichen symbionts [12, 13]. However, inducible mechanisms are also present, some similar to those observed in land plants whereas others seem to be exclusive to *T. gelatinosa.* In this alga, the presence of numerous and diverse desiccation-related proteins (DRPs) whose function is unknown is the most remarkable inducible mechanism. More recently, a proteomic approach to the extracellular polymeric substances (EPS) of the lichen microalgae TR9 and Csol subjected to cyclic D/R also revealed the existence of numerous and diverse DRPs of unknown functions exclusive of each microalga. Such proteins seem to play a species-specific role in adaption to water deficiency according to the habitat features [14] and the symbiotic lifestyle [11] of each microalga. A common fact in transcriptomic/proteomic studies carried out with desiccation-tolerant algae and resurrection plants is that organisms are subjected to a single cycle of D/R. However, in nature, algae and resurrection plants are typically exposed to diurnal and/or seasonal D/R cycles during their lifetime. The main objective of this study is to explore the molecular mechanisms involved in the responses of lichen phycobionts to D/R in an experimental context much closer to natural D/R conditions. We subjected Csol cultures to four consecutive cycles of slow D/R to reach this goal, during which we comparatively analysed the changes that occurred in their transcriptomes and proteomes.

## Methods

### Microalgae Culture and D/R Treatments

*Coccomyxa simplex* (Csol) (*Coccomyxa solorina*, according to [15] since it was isolated from the lichen *Solorina saccata*) was obtained from the culture collection of algae at Goettingen University (strain 216–12). Axenic microalgae culture and D/R treatments were performed as described in [16]. Microalgae were grown on semi-solid Bold 3N medium (1.5% agar, pH 6.8) in a growth chamber maintained at 15 °C with light/dark cycles of 14 h (25 µmol PAR m^−2^ s^−1^)/10 h. All experiments were performed with 21-day-old cultures (in which cells grew exponentially, approximately 180 mg fresh weight per culture). Each D/R cycle consisted of 10 h dehydration at 55% relative humidity and slow air movement (during the light period) and 14 h rehydration in water vapour-saturated sealed Petri dishes (4 h in the light and 10 in darkness). Dehydration was performed in a phytotron (KK 115 Top + , Pol-Eko-Apparatus, Poland) with temperature, light, relative humidity and airspeed control. Under these dehydration conditions, which resemble those of the habitat of the lichen *Solorina saccata* (of which Csol is the phycobiont) [9], the relative water content of Csol cells decreases to 6.97% (0.21 g water/g dry weight) after 10 h (Supplementary Fig. S1). This water content is higher than the 0.1 g water/g dry weight considered a “desiccated state”, but it is the lowest water level that allows Csol cells to recover full metabolic activity after rehydration [16]. Therefore, we consider that Csol has reached a state of severe dehydration.

Three independent samples were obtained from control conditions (C), after severe dehydration (D) and rehydration (R) of the second and fourth D/R cycle, as follows: C (C_1, C_2 and C_3), 2D (2D_1, 2D_2 and 2D_3), 2R (2R_1, 2R_2 and 2R_3), 4D (4D_1, 4D_2 and 4D_3) and 4R (4R_1, 4R_2 and 4R_3).

### RNA-Sequencing, De Novo Transcriptome Assembly and Relative Abundance Estimates of Transcripts

#### RNA Isolation/Purification

RNA was extracted from each sample (half-microalgal culture, 50–100 mg fresh weight) following the protocol of Jones et al. [17]. Contaminating residual DNA was removed from total RNA by treating it with the DNAse I RNA-free kit (Invitrogen, CA, USA). The quality of isolated RNAs was checked on denaturing agarose gels. RNA was quantified using a NanoDrop ND-1000 TM spectrophotometer (Daemyung, Korea). The RNA extracted from the replicates prepared for each sample under control conditions (C) and after treatments (2D, 2R, 4D and 4R) was distributed in equimolar quantities. Total RNA sample quality control (RNA concentration, RIN value, 28S/18S and the fragment length distribution) was performed using Agilent 2100 Bioanalyzer (Agilent RNA 6000 Nano Kit). Additional analyses were performed with NanoDropTM to assess the purity of the RNA samples.

#### cDNA Library Preparation and Sequencing

The construction of cDNA libraries and RNA sequencing was carried out following standard protocols for the BGISEQ-500 sequencing platform at BGI Genomics Co., Ltd. Hong Kong, China.

#### Read Cleaning and De Novo Transcriptome Assembly

Raw sequencing reads were filtered by removing reads with adapters, reads in which unknown bases (N) are more than 5% and low-quality reads defined as the percentage of bases whose quality is lesser than 10 and greater than 20% in a read. Cleaned reads are available at the NCBI Sequence Read Archive (SRA) under accession numbers SRR19371575-89. The cleaned reads were used for a de novo transcriptome assembly using Trinity software package v2.0.6 [18, 19], allowing a minimum contig length of 150 bp. TIGR software [20] was used to perform gene family clustering and to obtain the final predicted transcripts. The predicted transcripts were divided into two types; one type was a cluster, whose prefix is CL with the cluster id after it. In one cluster, there are several predicted transcripts whose similarity is more than 70%. Another type was a singleton, whose prefix is Unigene.

#### Relative Abundance Estimates of Transcripts

All the clean reads were mapped on the predicted transcripts with Bowtie2 v2.2.5 [21]. The number of fragments per million kilobases (FPKM) for each predicted transcript in the three replicates for each studied condition was calculated with RSEM v1.2.12 [22] with default parameters (Supplementary File 1). The identification of differentially abundant transcripts between samples was performed with NOIseq [23] according to the following criteria: Fold change ≥ 2, or ≤  − 2and diverge probability ≥ 0.95 (Supplementary File 2).

### Protein Identification and Quantification by LC–MS/MS

#### Protein Isolation/Purification

The proteins were extracted from the replicates prepared for each of the three biologically independent samples under control conditions (C) and after two (2D and 2R) and four (4D and 4R) D/R cycles. Total cell proteins of each sample were precipitated by incubation with 10% trichloroacetic acid at 4 °C for 30 min, followed by centrifugation, resuspension in 50 µl ultra-pure water and second precipitation with cold pure acetone. After removing acetone, proteins (45 µg) were resuspended in 50 μl 8 M urea, sequentially treated with 10 mM dithiothreitol and 55 mM iodoacetamide and digested with 1 µg recombinant trypsin (Roche). The peptides obtained were acidified, desalted and concentrated by C18 reversed-phase chromatography on the tip (OMIX, Agilent technologies). The eluted peptides were dried under vacuum in speedVac (Savant) and resuspended in 12 μl of 2% acetonitrile and 0.1% formic acid.

#### Peptide Analysis by LC–MS/MS

Peptides were analysed by means of liquid nano-chromatography (nano Easy-nLC 1000, Thermo Scientific) coupled to a high-resolution mass spectrometer Q-Exactive HF (Thermo Scientific, Bremen, Germany). The nano-HPLC was coupled online to the nanoelectric source of the Q-exactive HF mass spectrometer. The peptides were analysed after entering by electrospray ionisation using the integrated tip in the analytical column. The peptides were detected in an m/z mass range of 350–2000 Da. MS/MS data were acquired in the data-dependent acquisition (DDA) mode of the MS.

#### Construction of a Customised Protein Database

The predicted transcript sequences in the transcriptomic approach were used to predict coding regions with TransDecoder (https://github.com/TransDecoder/TransDecoder/wiki). The translated predicted coding regions rendered a set of protein sequences used for peptide identification and protein inference in further proteomic analyses. The transcript sequence coding for the identified proteins has been deposited at DDBJ/EMBL/GenBank under the accession GKAR00000000. The version described in this paper is the first version, GKAR01000000.

#### Identification of Peptides, Protein Inference and Quantification

The identification of the peptides and inference of proteins and their quantification were carried out using the Thermo Scientific™ Proteome Discoverer™ software. Summarising, the MS/MS data processing began with the recalibration of the masses using a quick search with Sequest HT against the customised protein sequence database derived from the RNA-seq data and alignments of the chromatograms of the samples. Subsequently, we aligned the retention times among the different samples analysed for the quantification of the precursor ions, considering the unique peptides and razor peptides (peptides shared between a group of proteins) present in at least 50% of replicas, regardless of the modified peptides. Finally, the total amount of protein in the different samples was normalised using the total abundance of all peptides. The protein ratio was calculated as the geometric median of the peptide group ratios. The statistical test of variance analysis (ANOVA) was used to estimate the probability that these measures are different among the three replicas to test whether the abundance of a protein varies among replicas. The observed differences were considered significant if the *p*-value obtained was below 0.05. The *p*-value was corrected by the [24] method to rule out the false positive changes estimated by the *p*-value for a given level of false discovery rate (FDR), obtaining an adjusted *p*-value, also called *q* value, which better controls the FDR.

### Functional Annotation of Predicted Proteins/Transcripts

Functional annotation of the predicted proteins/transcripts was performed using three functional databases (KOG, KEGG and Uniprot). KOG annotation was performed with WebMGA [25]. The KOG database includes Clusters of Orthologous Groups of proteins (COGs) predicted orthologs for 7 eukaryotic genomes, which we named KOGs after eukaryotic orthologous groups [26]. The updated version of the COGs for unicellular organisms and the eukaryotic KOGs is accessible at http://www.ncbi.nlm.nih.gov/COG/. KEGG annotation was performed with KofamKOALA, which is accessible at https://www.genome.jp/tools/kofamkoala/. The annotation of the predicted proteins/transcripts according to the UniprotKB database was performed with Blast2GO [27], available at http://www.blast2go.de. Uniprot is the manually annotated and reviewed database of the UniProt Knowledgebase (UniProtKB), available at https://www.uniprot.org/. For all annotations, the *e*-value threshold was set to 1e^−5^.

### Additional Bioinformatic Analyses

Predictions of subcellular location of proteins were performed with DeepLoc 1.0, which predicts the subcellular localization of eukaryotic proteins by differentiating between 10 different localizations [28]. A file containing all the experimentally identified protein sequences encoded by ORFs within transcripts was used as input, whose sequences are available at DDBJ/EMBL/GenBank under the accession GKAR00000000.

Hierarchical clustering of protein/transcript/sample data according to similar transcript/protein abundance changes in each condition compared to the control was performed with the Expression Heat Map Option of Heatmapper [30]. This option made it possible to display data in the form of a heatmap. A file containing nine columns and 316 rows in.txt format was uploaded as input. The first column included the names of the transcripts (whose sequences are available at DDBJ/EMBL/GenBank under the accession GKAR00000000) containing the ORFs coding for all the experimentally identified proteins. Rows included numerical values corresponding to log2FC values for transcripts and proteins considered to be differentially abundant in the studied conditions indicated in the first row (PROT_2D, RNA_2D, PROT_2R, RNA_2R, PROT_4D, RNA_4D, PROT_4R and RNA_4R) compared to the control. Only the names of transcripts and transcripts whose encoded proteins were considered to be differentially abundant were included in the first column of the file. Once the data file was uploaded, hierarchical clustering was performed, resulting in dendrograms which could be viewed in Heatmapper’s main viewer window by selecting the Row Dendrogram and Column Dendrogram viewing options. The Pearson correlation coefficient with average linkage was the distance measurement method selected to perform the hierarchical clustering. Clustering was applied to both rows and columns. A list of Uniprot IDs of proteins belonging to each resulting cluster was used as input data for further gene ontology enrichment analyses. Gene ontology enrichment and graphical visualisation of enrichment results and gene characteristics were performed with ShinyGO v0.76.3 http://bioinformatics.sdstate.edu/go/ [29] with an FDR cutoff of 0.05. Genes were mapped to KEGG pathways with the “KEGG Mapper—Search Pathway” tool [31] using Reference search mode and KEGG identifiers for functional orthologs as a query. General manipulation of sequences was performed with Geneious Prime 2021.1.1. and 2022.1.1.

## Results

### Comprehensive Proteome Profiling and Their Coding Transcripts

**Fig. 1:**
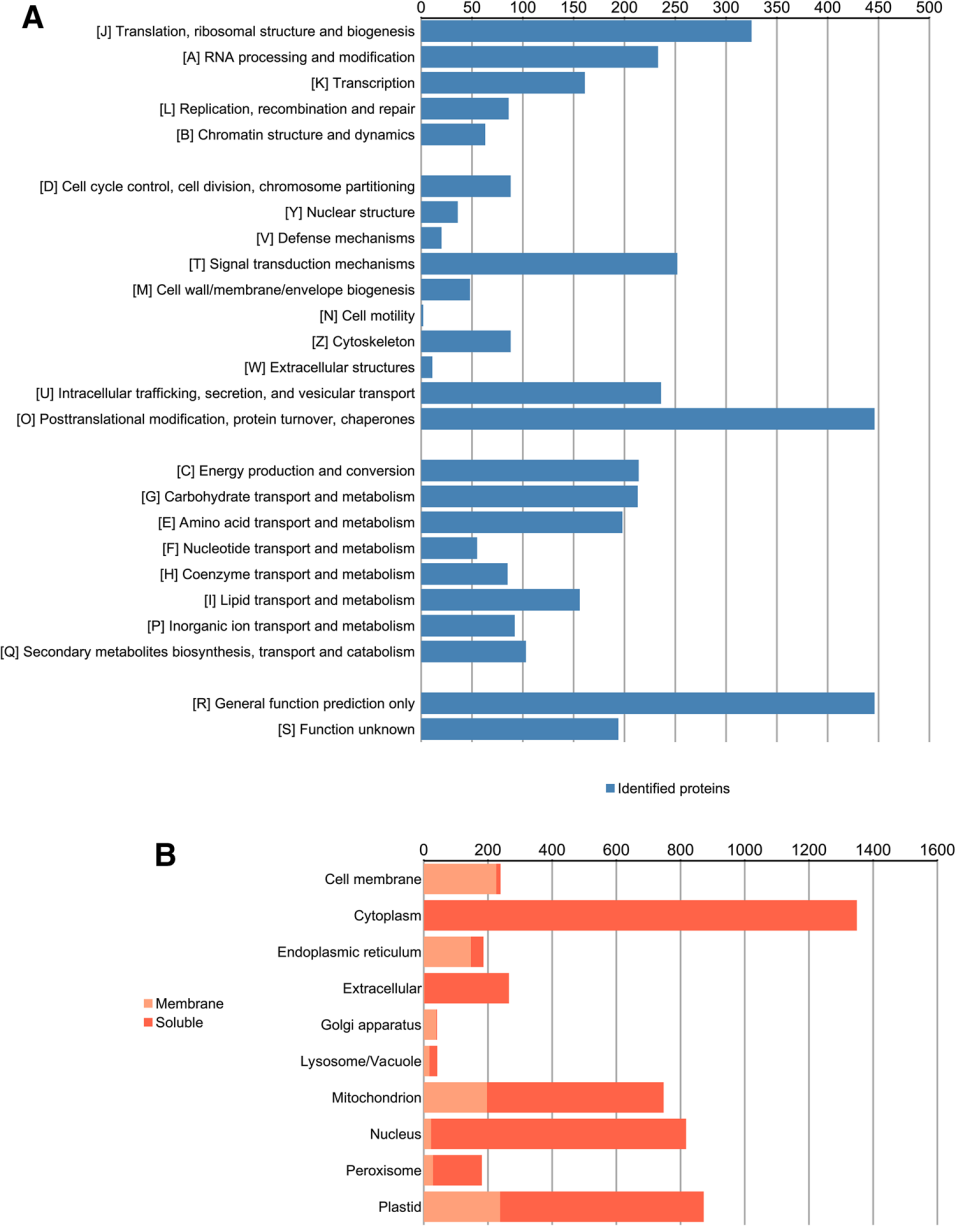
Functional classification and subcellular localization of Csol proteins. **A** KOG functional classification of proteins. Horizontal-axis represents the number of predicted proteins, and the vertical axis represents KOG categories. **B** Predicted subcellular localization of proteins. Horizontal-axis represents the number of predicted proteins, and the vertical axis represents subcellular compartments

In the present study, Csol cultures were subjected to four consecutive cycles of severe D/R. Afterwards, changes in the transcriptome and proteome were analysed to study their response under more natural conditions. Firstly, a reference transcriptome was obtained after sequencing the cDNA libraries from the same experimental conditions (C, 2D, 2R, 4D and 4R, in triplicates) used in posterior differential transcriptomic/proteomic analyses on the BGISEQ-500 platform. The resulting high-quality sequencing reads (Table S1) were assembled into transcripts with Trinity (Table S2), which were clustered with TIGR into gene families to obtain final predicted transcripts (Table S3). A total of 98,977 high-quality predicted transcripts with a total length of 316,703,266 bp, an average length of 3199 bp, an N50 of 4671 bp and a GC content of 54.69% (Table S4) were obtained. This reference transcriptome was used to construct a customised protein database, which was used for protein identification and relative quantification of the peptides as described in the “Methods” section. As a result, a total of 4779 proteins were identified (Supplementary File 3) and functionally annotated using KEGG, KOG and Uniprot databases (Supplementary File 4). Protein classification based on the COG code descriptions indicated the existence of at least 3657 (76.52%) proteins of known functions (Fig. 1A). About half of the identified proteins (2343) were involved in cellular processes and signalling (25.67%) and metabolism (23.35%). The prediction of protein subcellular localization with DeepLoc (Fig. 1B) revealed that 1349 proteins (28.23%) were cytoplasmic. A similar number of proteins were distributed among plastid (872 proteins, 18.25%), nucleus (817 proteins, 17.10%) and mitochondria (747 proteins, 15.63%). The remaining proteins (954 proteins, 20%) were distributed among extracellular space, cell membrane, endoplasmic reticulum, peroxisome, vacuole and Golgi apparatus.

### Protein and Transcript Profiles in Response to Cyclic D/R

**Fig. 2:**
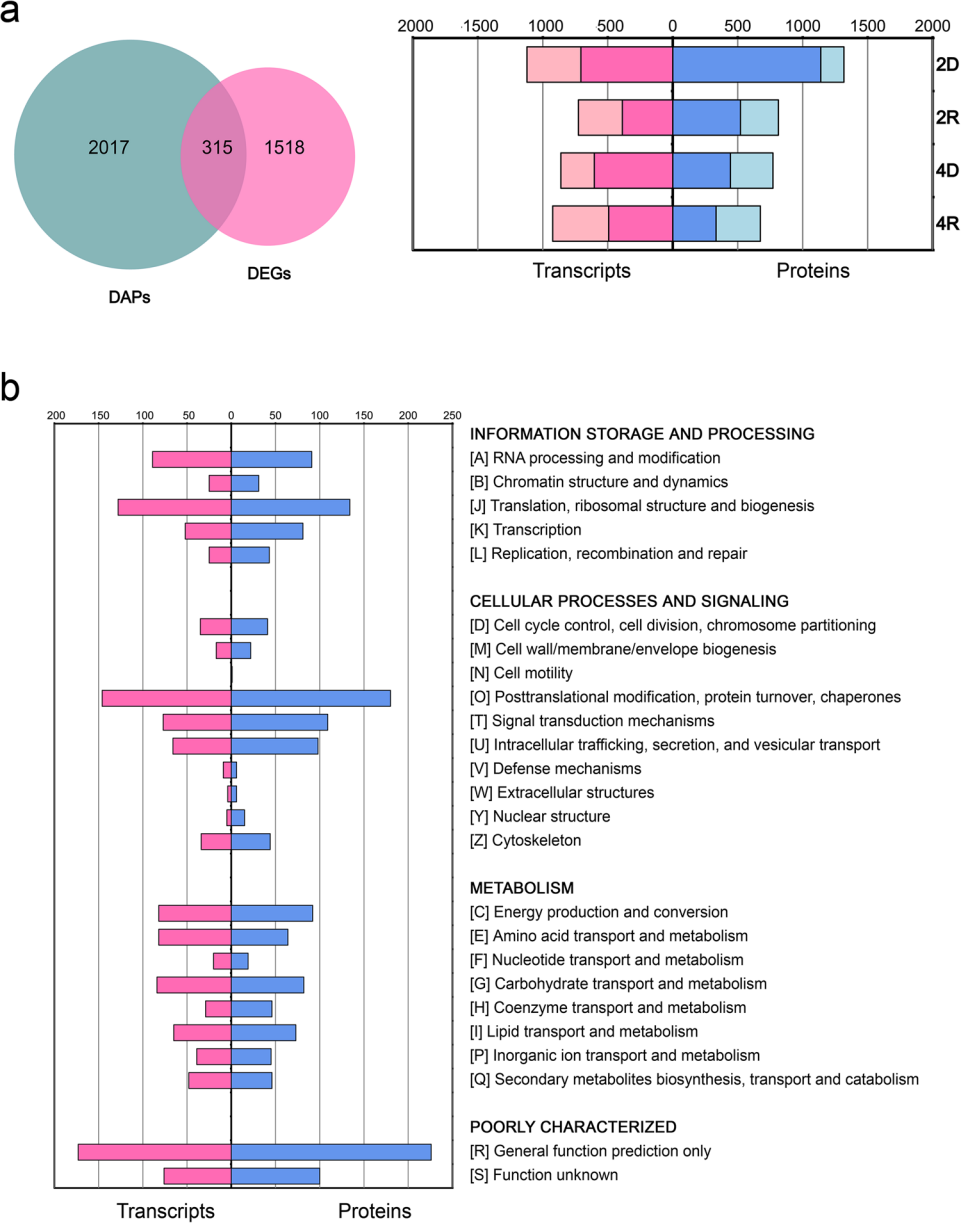
Distribution of DAPs/DATs in Csol under D/R after two and four D/R cycles. **a** Venn graph representing the association between DATs and DAPs members and a histogram representing the number of DAPs/DATs in each condition compared with control conditions and their expression trend. Decreasing and increasing transcripts or proteins are indicated in dark and light colours, respectively. **b** KOG functional classification of DAPs/DATs. Horizontal-axis represents the number of predicted DAPs/DATs and the vertical axis represents KOG categories

Estimations of protein abundance revealed the presence of 2332 proteins with statistically significant differences in abundance in some of the D/R treatments (2D, 2R, 4D and 4R) compared to control conditions (differentially abundant proteins, DAPs; *p*-value < 0.05 and fold change > 2.0) (Figs. 2a and S2). We also estimated the differences of the corresponding transcript levels in the same conditions (differentially abundant transcripts, DATs: Prob > 0.95 and fold change > 2.0) (Figs. 2a and S3), resulting in 1833 DATs. There were more decreasing than increasing proteins and transcripts in all the studied conditions relative to the hydrated control, except in 4R, with a slightly higher abundance of increasing rather than decreasing proteins. Regarding proteins, dehydrated cells showed ratios of 6.40 and 1.36 of decreasing/increasing proteins for 2D and 4D, respectively, whereas rehydrated showed ratios of 1.78 and 0.98 of decreasing/increasing proteins for 2R and 4R, respectively. Concerning transcripts, dehydrated cells showed ratios of 1.70 and 2.35 of decreasing/increasing transcripts for 2D and 4D, respectively, while rehydrated ones showed ratios of 1.15 and 1.14 of decreasing/increasing transcripts for 2R and 4R, respectively.

To analyse the main functions of DATs/DAPs, we searched functional orthologs (FOs) among the annotated DAPs/DATs by using the appropriate BLAST tools by performing searches against the euKaryotic Orthologous Group (KOG) database with an *E*-value threshold of 1e^−5^ (Fig. 2b). Functions related to translation, ribosomal structure and biogenesis, post-translational modifications, protein turnover and chaperones showed the highest proportion of DAPs and DATs, followed by transcription and RNA processing. Metabolic functions, including energy production and conversion, transport and metabolism of carbohydrates, lipids and amino acids, were also represented as well as signal transduction mechanisms. A total of 360 proteins of unknown function were also present among the 2332 DAPs (15.43% of DAPs). Similarly, a total of 208 DATs coding for proteins of unknown function were present among the 1833 DATs (11.35% of DATs).

**Fig. 3:**
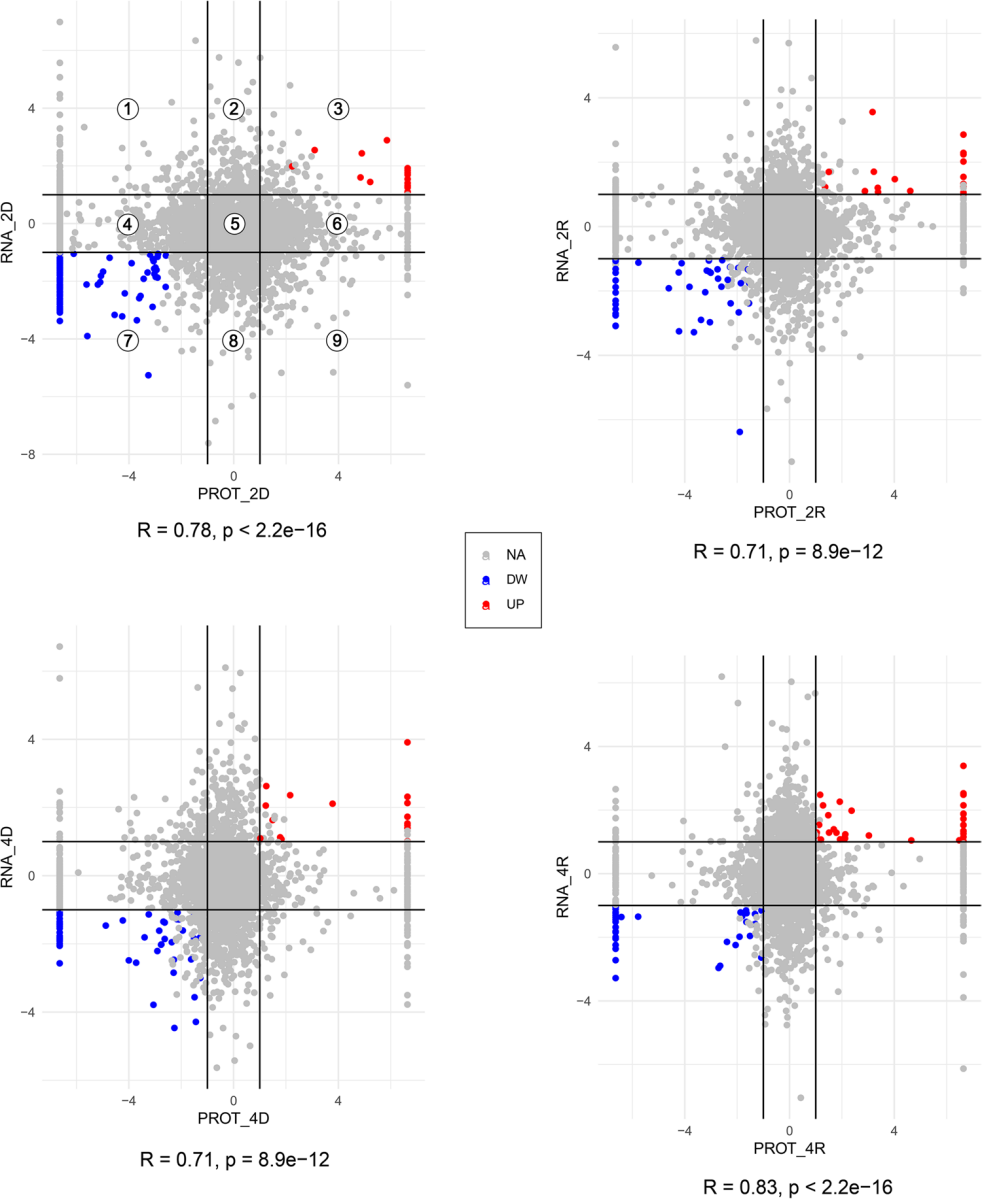
Nine-quadrant diagram of transcriptome and proteome association analysis. The horizontal coordinates indicate DAPs (log2FC ≤  − 1.0 and log2FC ≥ 1.0, *p*-value < 0.05) and the vertical coordinates indicate DATs (log2FC ≤  − 1.0 and log2FC ≥ 1.0, prob > 0.95). Differential expression values obtained using RNA-Seq and Protein-Seq were used as data points and correlated by a nine-quadrant. Coloured spots within quadrants (3) and (7) represent genes with a differential abundance of both protein and mRNA that followed the same trend; (1) and (9) indicate genes with a differential abundance of both protein and mRNA but following opposite trends; (4) and (6) indicate genes with a differential abundance of protein and no differential abundance of mRNA; (2) and (8) indicate genes with a differential abundance of mRNA and no differential abundance of protein; (5) indicates genes with no difference in protein and mRNA expression. Pearson’s correlation coefficients (R) and *p*-values (p) for each condition are indicated below each diagram

Despite the similar functional profiles of DAPs and DATs shown in Fig. 2b, the expression data of DAPs and DATs showed a low correlation. A relatively scarce number of DAPs/DATs pairs showed congruent expression data (Figs. 3 and S3): 165 DAPs (147 decreasing and 18 increasing proteins) for 2D, 64 DAPs (44 decreasing and 20 increasing proteins) for 2R, 69 DAPs (53 decreasing and 16 increasing proteins) for 4D, 71 DAPs (36 decreasing and 35 increasing proteins) for 4R. Interestingly, the correlation analyses on differentially abundant proteins/genes across all four studied conditions revealed a high correlation within dehydration or rehydration treatments and cycles for both proteins (Fig. S4) and transcripts (Fig. S5) in these sets of proteins/genes.

### Functional Analysis of Differentially Abundant Proteins Throughout Cyclic D/R

**Fig. 4:**
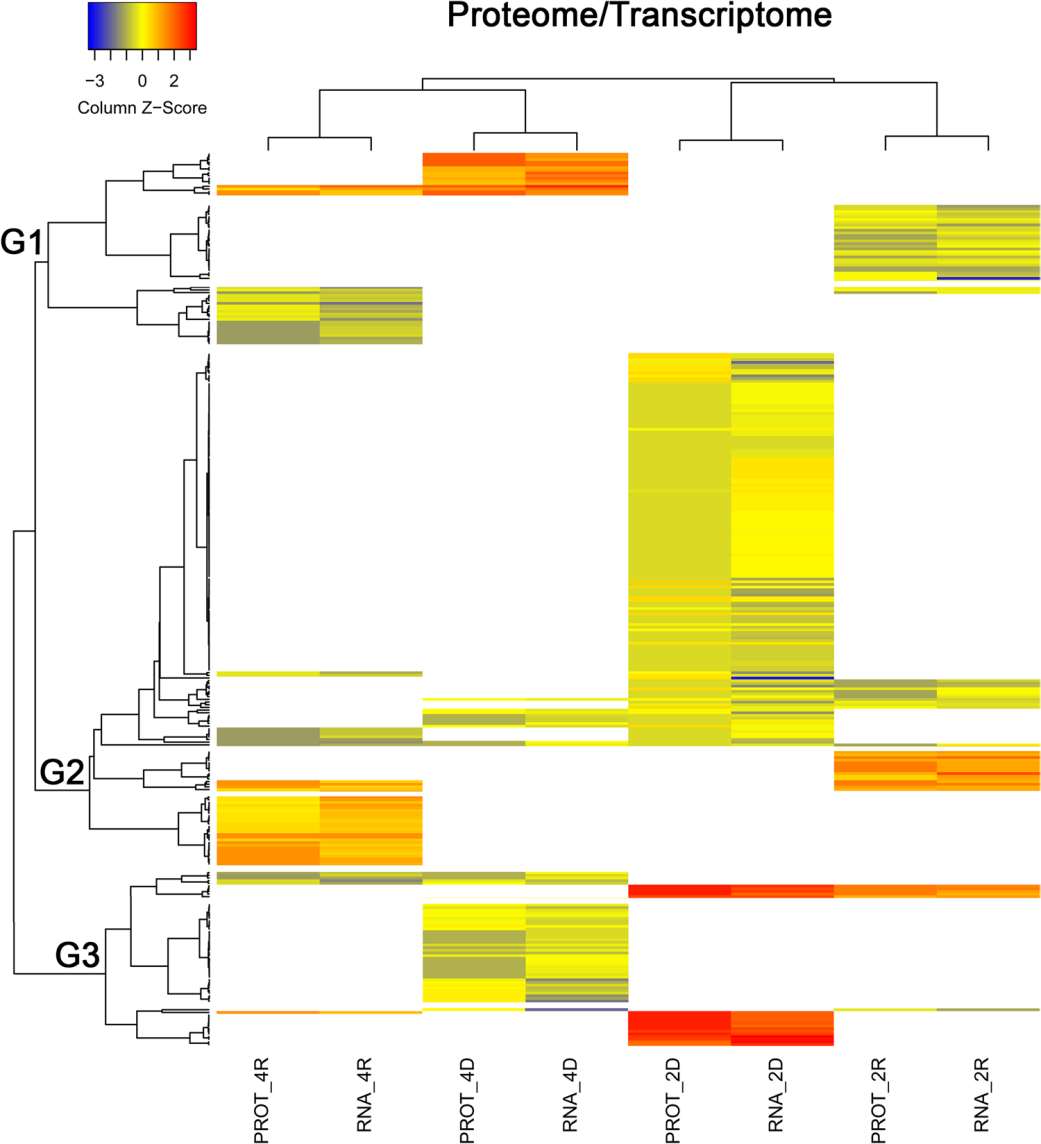
Heatmap of DAPs/DATs after two and four D/R cycles. The colours in the scale (blue-green (low) and red–orange (high) represent the normalised expression levels of DAPs/DATs, white represents missing values. Hierarchical clustering was performed with average linkage and Pearson correlation coefficient, grouping data by similar expression levels

**Fig. 5:**
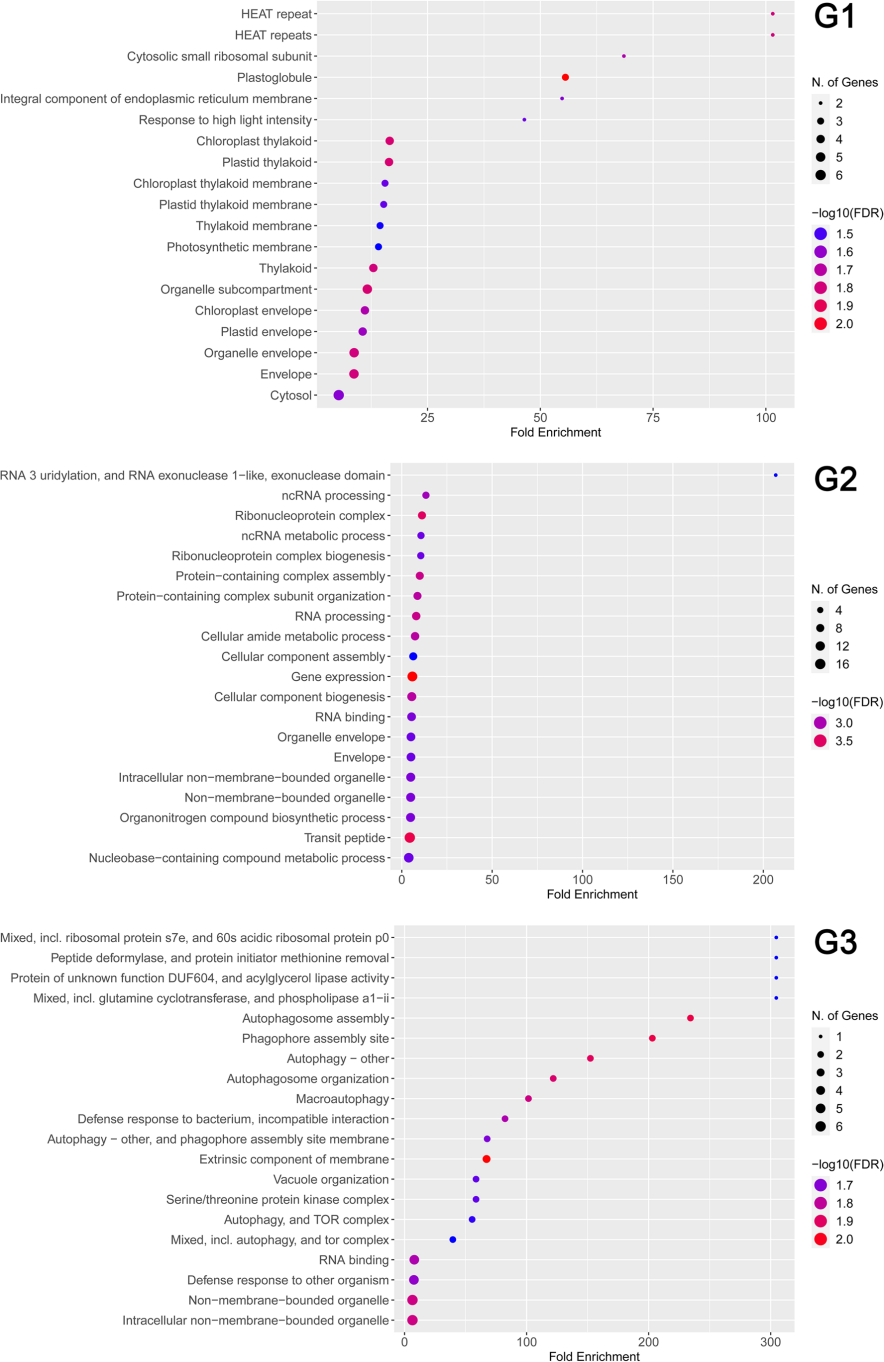
Dot plot of Gene Ontology (GO) enrichment analysis results obtained with the ShinyGO v0.61 application. The points in each dot plot are sized by the proportion of all proteins within the cluster annotated with the corresponding term and coloured by enrichment confidence (FDR). For the complete results of the GO enrichment analysis, see Supplementary File S2. G1, G2 and G3 correspond to the clusters of Fig. 4

We focused our study on the set of 315 DAPs/DATs whose changes in abundance were congruent with each other. Firstly, a hierarchical clustering (with average linkage and Pearson correlation coefficient) grouping data by similar abundance variations was performed on these 315 DAP/DAT pairs (Fig. 4) to examine the changes in the abundance of transcripts and their corresponding proteins throughout cyclic D/R. The analysed DAP/DAT pairs were grouped into three major clusters (i.e. G1–G3) with distinct abundance patterns. All three clusters showed analogous changes in transcript and protein abundance. This result aligned with Pearson’s correlation analyses, indicating a direct link between changes in abundance patterns of transcripts and their encoded proteins. The gene ontology enrichment analysis of transcripts/proteins included in each of the three clusters (Fig. 5 and Supplementary File 5) revealed that terms related to chloroplasts and response to high light intensity were enriched in G1. Terms related to RNA metabolism and protein-containing complex assembly were enriched in G2. Terms related to autophagy and defence response are mainly enriched in G3.

A more in-depth analysis of DATs related to information storage and processing (Fig. S6), metabolism, cellular processes and signalling (Fig. S7) was performed by searching functional orthologs (FOs). A number of proteins related to gene expression were differentially abundant in response to cyclic D/R (Fig. S6). Several proteins associated with chromosomes, involved in messenger RNA, ribosome and transfer RNA biogenesis, related to protein processing/degradation and ribosomal proteins were mainly decreased in dehydrated cells resulting in a generalised silencing of gene expression. The few proteins that were upregulated were generally found in rehydrated cells. The generalised decrease in transcripts seems to be accompanied by a similar reduction in basal metabolic activities in dehydrated cells (Fig. S7). Generally, enzymes related to carbon fixation, oxidative phosphorylation, starch and sucrose metabolism, nitrogen metabolism and glycerophospholipid metabolism, among others, were decreased in dehydrated cells and/or eventually increased in rehydrated cells. Decreasing these enzymes in dehydrated cells is consistent with an energy-saving survival strategy against dehydration. Increasing the same enzymes in rehydrated cells is consistent with re-establishing metabolic activity. The PSII-associated protein psbS, involved in protecting the cell against photo-oxidative damage, increased in rehydrated cells after four D/R cycles compared with control cells.

Key antioxidant enzymes such as superoxide dismutase, glutathione reductase and catalase increased only after four D/R cycles in both dehydrated and rehydrated cells. A protein involved in the synthesis of raffinose also increased after four D/R cycles in rehydrated cells. Increasing in these proteins could be explained by their involvement in protective mechanisms against stress. Plant hormones and signal transduction elements seem to play an important role in DT (Fig. S7). Some DATs/DAPs are involved in response to ethylene (e.g. ethylene receptor [EC:2.7.13.-]) and abscisic acid (e.g. serine/threonine-protein kinase SRK2 [EC:2.7.11.1]). Other DAPs are involved in a variety of signalling pathways, including several protein kinases and phosphatases. A protein related to autophagy involved in plant nutrient recycling increased in dehydrated cells after two D/R cycles. A series of proteins involved in transporting various substances either increased or decreased in different conditions. Several chaperones and folding catalysts are also differentially abundant.

## Discussion

*Coccomyxa simplex* (Csol) is a chlorophyte symbiotically associated with the fungus *Solorina saccata* in lichen thalli. This lichen species usually grows in limestone crevices where it is subjected to slowly developed daily periods of drying during summer [32]. Csol is well adapted to this environment because it seems to provide both constitutive and inducible mechanisms to cope with D/R [9]. In this study, we investigate the impact of more than one D/R cycle on the transcript and protein profiles in Csol cells to reproduce natural conditions better, generally consisting of successive diurnal and/or seasonal D/R cycles. To the best of our knowledge, this is the first study to comparatively analyse changes in transcriptional and protein profile during cyclic dehydration in a lichen phycobiont. A total of 1833 transcripts and 2332 proteins increased or diminished in abundance after two and four D/R cycles relative to the hydrated control. It is well known that transcript/protein abundance does not indicate a direct causal relationship with gene transcription or protein synthesis but rather the balance of these activities, transcript/protein turnover and post-transcriptional/post-translational processes, which could be influenced by dehydration. For instance, in the desiccation-tolerant bryophyte *Tortula* *ruralis* mRNAs are sequestered in mRNPs during slow dehydration resulting in an apparent increase in transcript abundance as a result of the protection of the mRNAs from degradation [33]. However, qualitative and quantitative changes in the transcript and protein profiles are widely considered potent predictors of how organisms adjust their major life functions to environmental variations, including gene expression and protein synthesis. Previous studies in other desiccation-tolerant plants (e.g. the streptophyte alga *Klebsormidium crenulatum* [34], the moss *Bryum argenteum* [35] and the resurrection plants *Myrothamnus flabellifolia* [36] and *Sporobolus stapfianus* [37]) reported thousands of DATs after analysing transcriptome profiles in different conditions. At first glance, finding a high number of differentially abundant transcripts/proteins depending on the water availability in Csol suggests that inducible mechanisms in DT in this microalgal species play an important role. However, only 315 differentially abundant transcripts/proteins showed correlative variations indicating a precise and limited repertory of affected transcripts/proteins in response to D/R and, on the other hand, that changes in the transcriptional or protein profile should be analysed together to obtain a more accurate functional perspective. This finding is consistent with the considerable contribution of constitutive mechanisms to achieve DT in the lichen-forming microalga *Trebouxia gelatinosa* [11] and the monilophyte *Selaginella tamariscina* [38]).

In Csol, decreased transcripts/proteins are more abundant than upregulated ones in comparisons between dehydrated cells and control conditions after either two or four D/R cycles. A similar picture can be observed in some desiccation-tolerant plants (e.g. *Klebsormidium crenulatum* [34] and *Myrothamnus flabellifolia* [36]). However, in other desiccation-tolerant plants, upregulated genes are moderately more abundant than down-regulated ones (e.g. *T. gelatinosa* [11] and *S. tamariscina* [38]). This is also the case of highly desiccation-tolerant seeds, which reduce metabolic activity during maturation and change the use of carbon to accumulate protective substances. Overall, our results in Csol microalgae are compatible with a transient decrease in many functions and metabolic activities related to cellular growth due to a severe dehydration state and the concomitant increase in specific biological processes necessary to survive in dry conditions [39]. Accordingly, we found lesser quantities of down- and upregulated proteins/genes after four D/R cycles than after two cycles, particularly in severely dehydrated cells. These findings can be explained by an acclimation/priming process experienced by fully hydrated cells exposed to successive D/R cycles. Many cellular functions inhibited in the initial desiccation steps undergo a gradual reactivation to accommodate cyclic D/R conditions.

Previous studies indicate that desiccation-tolerant and desiccation-sensitive microalgae increase the products of common protective genes [40]. However, the main feature distinguishing desiccation-tolerant from desiccation-sensitive microalgae is a controlled decrease in transcripts/proteins related to growth, energy production and metabolism during desiccation. In this study, we observed both a controlled reduction of transcripts/proteins related to metabolism and cellular functions and increasing in transcripts/proteins related to protective genes/proteins during desiccation. The decrease of DAPs/DATs related to carbohydrate transport and metabolism is consistent with extensive adjustments in carbohydrate metabolism observed in Csol in response to dehydration in previous studies (e.g. [9, 41, 42]). Some of these adjustments affect the cell wall, which undergoes a marked remodelling of polysaccharides that could increase cell wall flexibility [42] and the extracellular polysaccharides loosely attached to the cell wall, modifying their hydrophilicity [42]. In addition, the increase in a DAP/DAT involved in polyol metabolism is consistent with the recent attribution of two main functionalities to polyols as respiratory substrates and as an adaptation to desiccation, which is crucial to the evolution of fungal symbionts [43]. An increase in raffinose-related transcripts has also recently been shown, which is affected by cyclic D/R with a more inducible response in Csol than in other lichen microalgae [44].

Both desiccation-tolerant and sensitive plants undergo a decrease in photosynthetic rates during drought. However, only desiccation-tolerant plants are able to completely recover photosynthesis after rehydration (reviewed in [45]). In this study, only a few transcripts/proteins related to photosynthesis are differentially abundant. This observation is compatible with conserving the photosynthetic proteins during dehydration in homoiochlorophyllous desiccation-tolerant plants. Previous results indicated that when Csol is subjected to daily slow dehydration cycles, this microalga can fully recover its photosynthetic rates after at least four D/R cycles [16]. Plants possess mechanisms to avoid photo-oxidative damage caused by the absorption of light when photosynthesis is blocked, such as by dissipating excess light energy through non-photochemical quenching in which protein psbS plays an important role [46] and by diminishing the size of the light-harvesting complex of PSI and PSII thus reducing light absorption [47]. In our study, the psbS protein increased, and two proteins of the light-harvesting complex I and II diminished in rehydrated cells after four D/R cycles (Fig. S7).

Differential dehydration tolerance across green microalgal species, assessed by photosynthetic efficiency during D/R cycles, was related to differential intracellular ROS balance [48]. In lichens, scavenging of ROS can be carried out by antioxidant enzymes, which are basically superoxide dismutases, catalases, glutathione reductase and peroxidases [49-51]. Previous studies indicated an increase in the transcript levels for a superoxide dismutase in dehydrated cells of the lichen microalgae Csol and *Trebouxia* sp. TR9 [16] and *Trebouxia gelatinosa* [11]. In this study, several antioxidant enzymes, including superoxide dismutase, catalase and glutathione reductase, increased in either dehydrated or rehydrated cells after four D/R cycles compared to control conditions. Recent results have demonstrated that the ascorbate–glutathione cycle plays an important role in overcoming oxidative stress during drying in the resurrection plant *Haberlea rhodopensis* [52]. In Csol, the increase in transcripts/proteins encoding a glutathione reductase, which is a key component of the ascorbate recycling system and is involved in antioxidant protection [53], is consistent with the observed increase of ascorbic acid during dehydration in Csol [9].

Stress accommodation mechanisms must be essentially regulated at the gene level to allow either silencing or expression of appropriate genes. Regulation of gene expression can involve chromatin remodelling accompanied by epigenetic changes, including histone modification and DNA methylation. Epigenetic marks seem to contribute to a kind of stress memory, which enables plants to respond more effectively and efficiently to recurring stresses [54]. In Csol, a number of DAPs are associated with chromosomes and may contribute to chromatin remodelling and epigenetic changes, which can either activate or deactivate gene expression (Fig. S6). On the other hand, transcription factors directly regulate the expression of genes by interacting with specific cis-elements in their promoter region. In this study, several transcription factors are shown to change their amount in response to cyclic D/R. Gene expression is also regulated by several post-transcriptional, translational and post-translational processes that act as part of the defence response of plants during environmental stresses, including desiccation in desiccation-tolerant plants [33, 55]. This response partly depends on the coordinate activity of transcription factors and phytohormone signalling elements [56]. For instance, transcription factor E2FB, which has recently been involved in regulating DNA reparation [57], is increased during the second D/R cycle.

In contrast, at the same time, TGE11 and ethylene-responsive transcription factor 12, which are transcriptional repressors of abscisic acid and ethylene responses [58, 59], respectively, are decreased (Fig. S7). It is possible that this strategy of “repressing defenders’ repressors” could enhance the effectiveness of constitutive defence mechanisms in organisms exposed to rapid changes in water status. Within post-translational regulation and stress response, phosphorylation of proteins by kinases modulates protein function, including transcription factors, which can be stimulated or repressed along successive D/R cycles, helping microalgae to thrive with changes in water availability in their natural habitats [60]. In our study, the quantities of several transcripts/proteins related to phytohormones and additional signalling elements such as ethylene receptor, protein kinases and phosphatases are affected by cyclic D/R (Fig. S7).

Plant autophagy is essential for eliminating damaged cellular components. Various autophagy pathways have specific roles during plant development and stress response [61]. Cyclic D/R produces ongoing cell damage, requiring selective recycling of cellular components. This study also suggests that autophagy has an important role in the dehydration-tolerance of Csol since many transcripts/proteins related to this biological process (peptidases, transporters) were differentially abundant in response to cyclic D/R.

### Conclusion and Perspectives

Exposure of dehydration-tolerant algae such as Csol to successive cycles of severe D/R causes major changes in the transcriptional and protein profile, which, in turn, change over time and are consistent with a profound slowing in major metabolic activities and readiness to tolerate water loss, especially in the initial cycles. Thereafter, the observed lesser quantities of down- and upregulated proteins/genes suggest an acclimation to successive D/R cycles and a gradual reactivation to accommodate cyclic D/R conditions. These changes include an extensive decrease in transcript and protein abundance during dehydration involved in gene expression, metabolism, substance transport, signalling and folding catalysis and increasing transcripts/proteins related to antioxidant defence, polyol metabolism and autophagy.

The results of this work give an overview of the mechanisms that play a major part in tolerating severe dehydration of Csol and perhaps of other dehydration-tolerant microalgae. At the same time, the results suggest that new elements/strategies of this complex phenomenon can be addressed in further studies. The high proportion of genes of unknown functions related to DT is noteworthy, which have also been found in other lichen-forming chlorophytes (e.g. *T. gelatinosa* [11], *Asterochloris* sp. [62]) and in non-Chlorophyta algae (e.g. [63]). Therefore, the major challenge is to elucidate the functions in which the expression products of these genes are involved, which may lead to the discovery of new DT elements. It would also be interesting to study the interplay of chromatin remodellers, transcription factors and hormonal signalling, as well as the possible interplay between constitutive and inducible defence mechanisms, as suggested in our study. To our knowledge, these topics concerning dehydration tolerance in microalgae have not been addressed.

## Supplementary Information

Below is the link to the electronic supplementary material.

Supplementary file1 (DOCX 6 KB)

Supplementary file2 (TIF 2856 KB)

Supplementary file3 (TIF 2708 KB)

Supplementary file4 (XLSX 5617 KB)

Supplementary file5 (XLSX 6773 KB)

Supplementary file6 (XLSX 1201 KB)

Supplementary file7 (XLSX 645 KB)

Supplementary file8 (XLSX 12 KB)

Supplementary file9 (DOCX 6 KB)

Supplementary file10 (DOCX 6 KB)

Supplementary file11 (DOCX 7 KB)

Supplementary file12 (TIF 538 KB)

Supplementary file13 (TIF 918 KB)

Supplementary file14 (TIF 1001 KB)

Supplementary file15 (TIF 1722 KB)

Supplementary file16 (TIF 1463 KB)

## Data Availability

The datasets generated during and/or analysed during the current study are available in the attached supplementary files. Any additional information is available from the corresponding author upon reasonable request. Molecular data are available in BioProject ID PRJNA841810. The transcript sequence coding for the identified proteins has been deposited at DDBJ/EMBL/GenBank under the accession GKAR00000000.

## Code Availability

This study does not include any software application or custom code.
