# Supplementary material for: Comparative Transcriptomic and Proteomic Analyses Provide New Insights into the Tolerance to Cyclic Dehydration in a Lichen Phycobiont
Source: Microb Ecol. 2023 Apr 11;86(3):1725–39. doi: 10.1007/s00248-023-02213-x (PMC10497648; doi:10.1007/s00248-023-02213-x)
Supplement: Supplementary file 1 — Supplementary file1 (DOCX 6 KB) [file 248_2023_2213_MOESM1_ESM.docx]

**Table S1:** Filtered reads quality metrics.

| **Sample** | **Total Raw Reads(M)** | **Total Filtered Reads(M)** | **Total Filtered Bases(Gb)** | **Filtered Reads Q20(%)** | **Filtered Reads Q30(%)** | **Filtered Reads Ratio(%)** |
| --- | --- | --- | --- | --- | --- | --- |
| **C1** | 116 | 111.47 | 11.15 | 95.75 | 85.5 | 96.1 |
| **C2** | 115.62 | 111.42 | 11.14 | 96.23 | 86.9 | 96.37 |
| **C3** | 117.88 | 113.37 | 11.34 | 96.16 | 86.69 | 96.18 |
| **2D1** | 126.9 | 122.64 | 12.26 | 96.66 | 88.26 | 96.64 |
| **2D2** | 121.92 | 118.03 | 11.8 | 96.88 | 88.88 | 96.81 |
| **2D3** | 124.4 | 119.97 | 12 | 96.72 | 88.56 | 96.43 |
| **2R1** | 124.4 | 119.95 | 12 | 96.62 | 88.23 | 96.42 |
| **2R2** | 124.41 | 119.22 | 11.92 | 96.45 | 88.13 | 95.83 |
| **2R3** | 124.4 | 120.51 | 12.05 | 96.63 | 88.29 | 96.87 |
| **4D1** | 115.54 | 111.39 | 11.14 | 96.46 | 87.65 | 96.41 |
| **4D2** | 115.63 | 111.84 | 11.18 | 96.66 | 88.03 | 96.73 |
| **4D3** | 118.1 | 113.4 | 11.34 | 96.09 | 86.62 | 96.02 |
| **4R1** | 115.53 | 111.62 | 11.16 | 96.24 | 86.86 | 96.61 |
| **4R2** | 118.07 | 113.4 | 11.34 | 95.98 | 86.31 | 96.05 |
| **4R3** | 117.69 | 111.38 | 11.14 | 94.88 | 83.41 | 94.64 |

Samples: replicates for control conditions (C1-C3), replicates for desiccation conditions after four (4D1-4D3) and two (2D1-2D3) D/R cycles, replicates for rehydration conditions after four (4R1-4R3) and two (2R1-2R3) D/R cycles.

Total Raw Reads(Mb): reads amount before filtering.

Total Filtered Reads(Mb): reads amount after filtering.

Total Filtered Bases(Gb): total base amount after filtering.

Filtered Reads Q20 (%): rate of bases which quality is greater than 20 value in Filtered reads.

Filtered Reads Q30( %): rate of bases which quality is greater than 30 value in Filtered reads.

Filtered Reads Ratio (%): ratio of the amount of Filtered reads.
