# Supplementary figures and images for: Comparative Transcriptomic and Proteomic Analyses Provide New Insights into the Tolerance to Cyclic Dehydration in a Lichen Phycobiont
Source: Microb Ecol. 2023 Apr 11;86(3):1725–39. doi: 10.1007/s00248-023-02213-x (PMC10497648; doi:10.1007/s00248-023-02213-x)

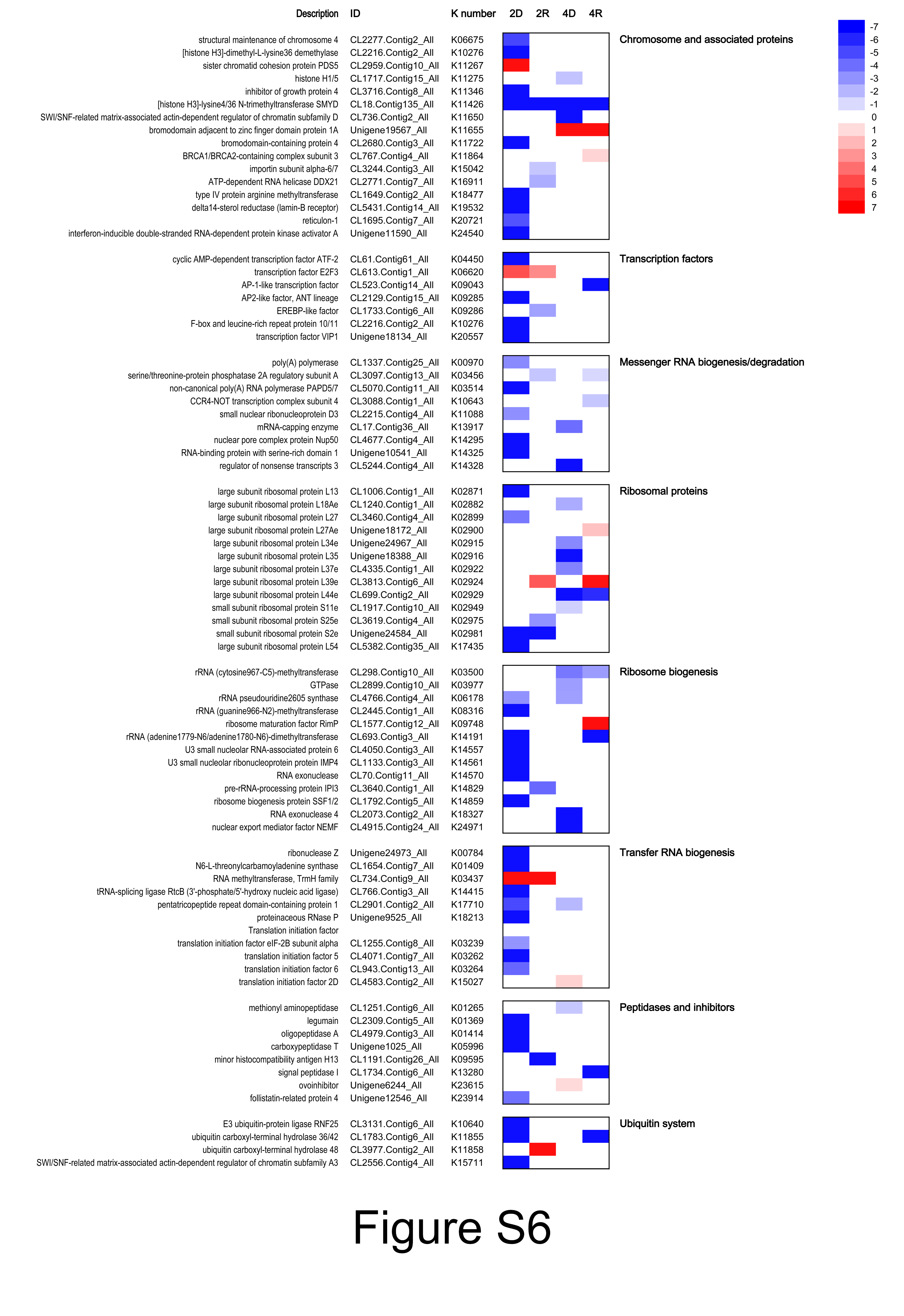

Supplement: Supplementary file 2 — Supplementary file2 (TIF 2856 KB) [file 248_2023_2213_MOESM2_ESM.tif]

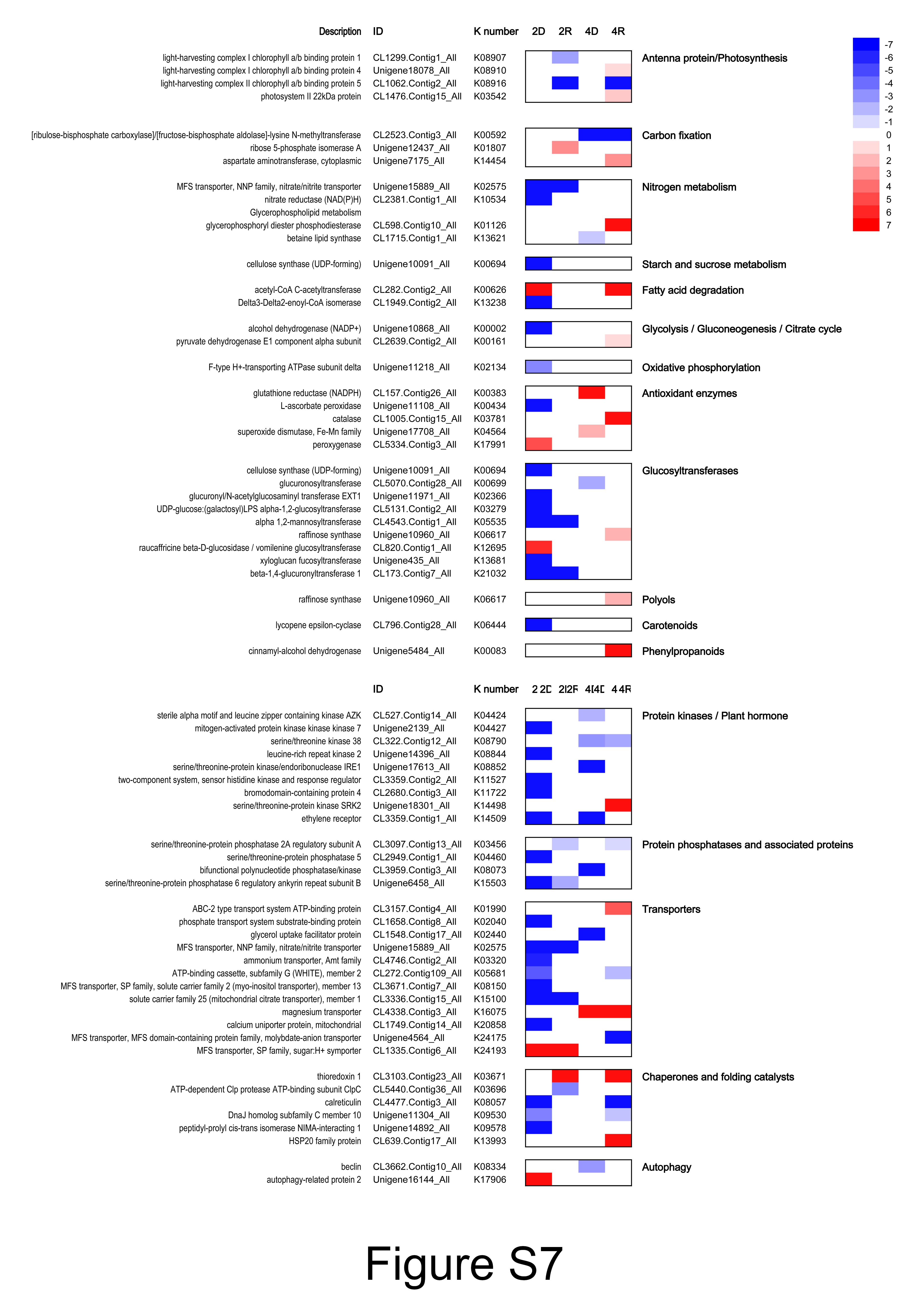

Supplement: Supplementary file 3 — Supplementary file3 (TIF 2708 KB) [file 248_2023_2213_MOESM3_ESM.tif]

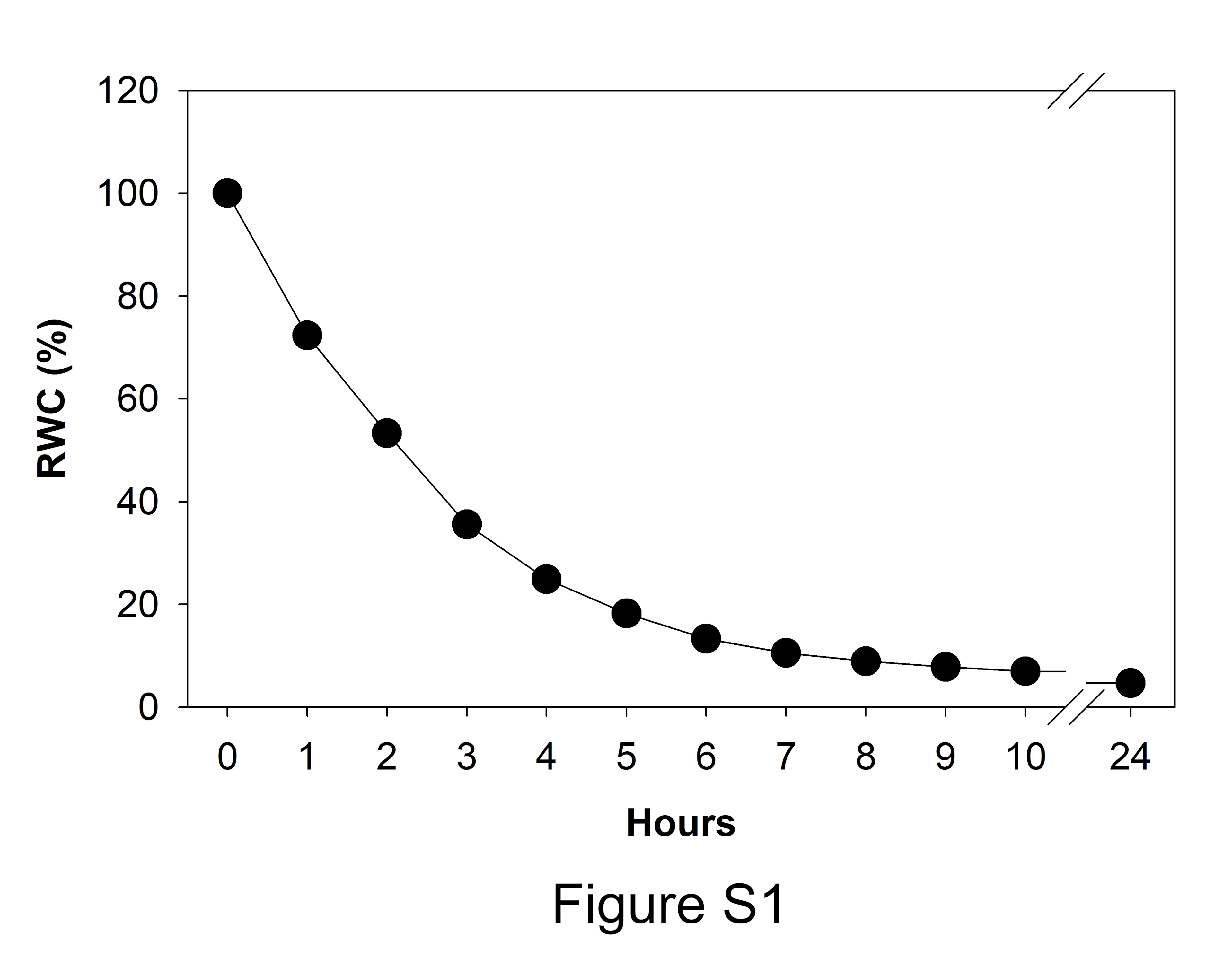

Supplement: Supplementary file 12 — Supplementary file12 (TIF 538 KB) [file 248_2023_2213_MOESM12_ESM.tif]

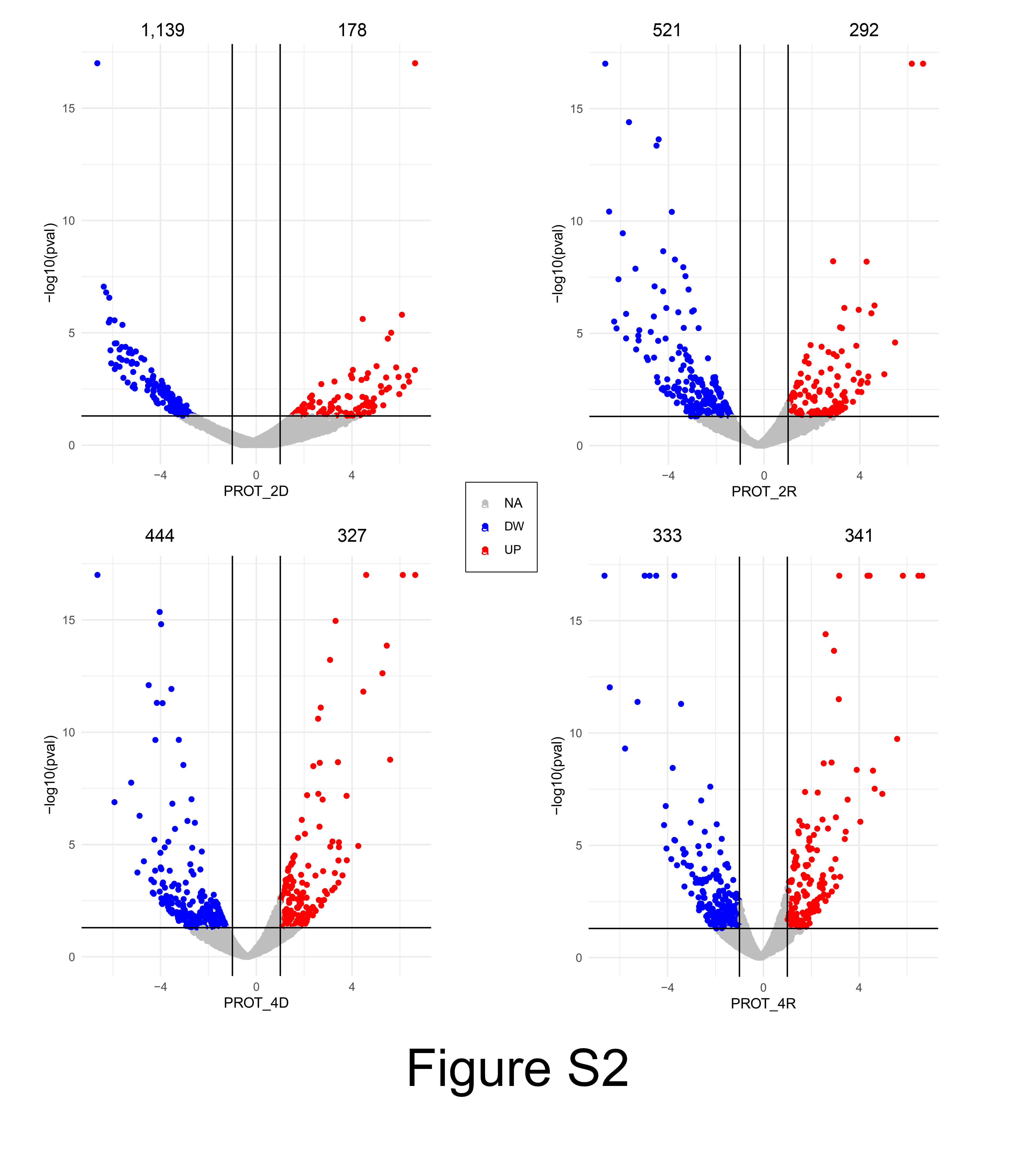

Supplement: Supplementary file 13 — Supplementary file13 (TIF 918 KB) [file 248_2023_2213_MOESM13_ESM.tif]

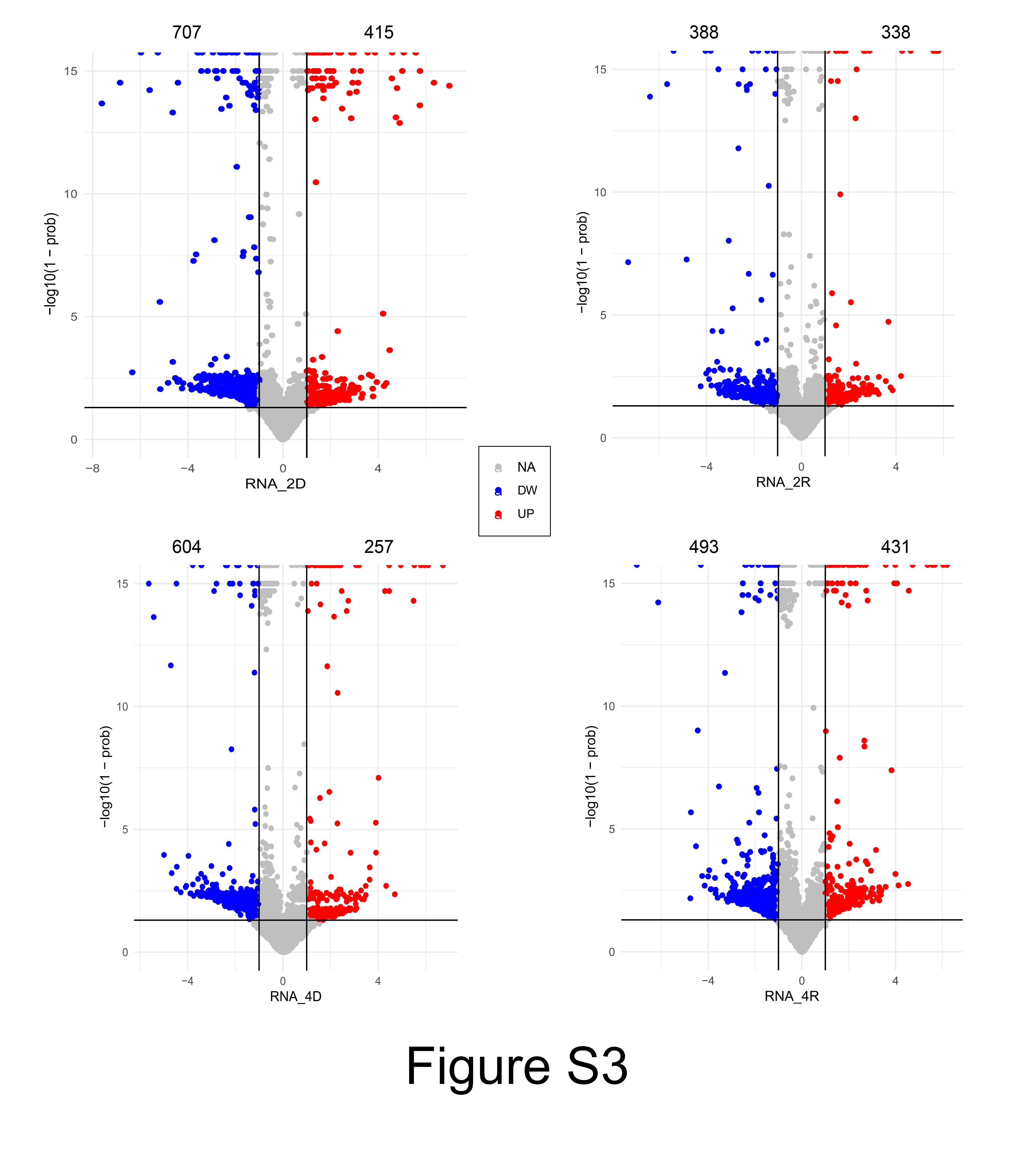

Supplement: Supplementary file 14 — Supplementary file14 (TIF 1001 KB) [file 248_2023_2213_MOESM14_ESM.tif]

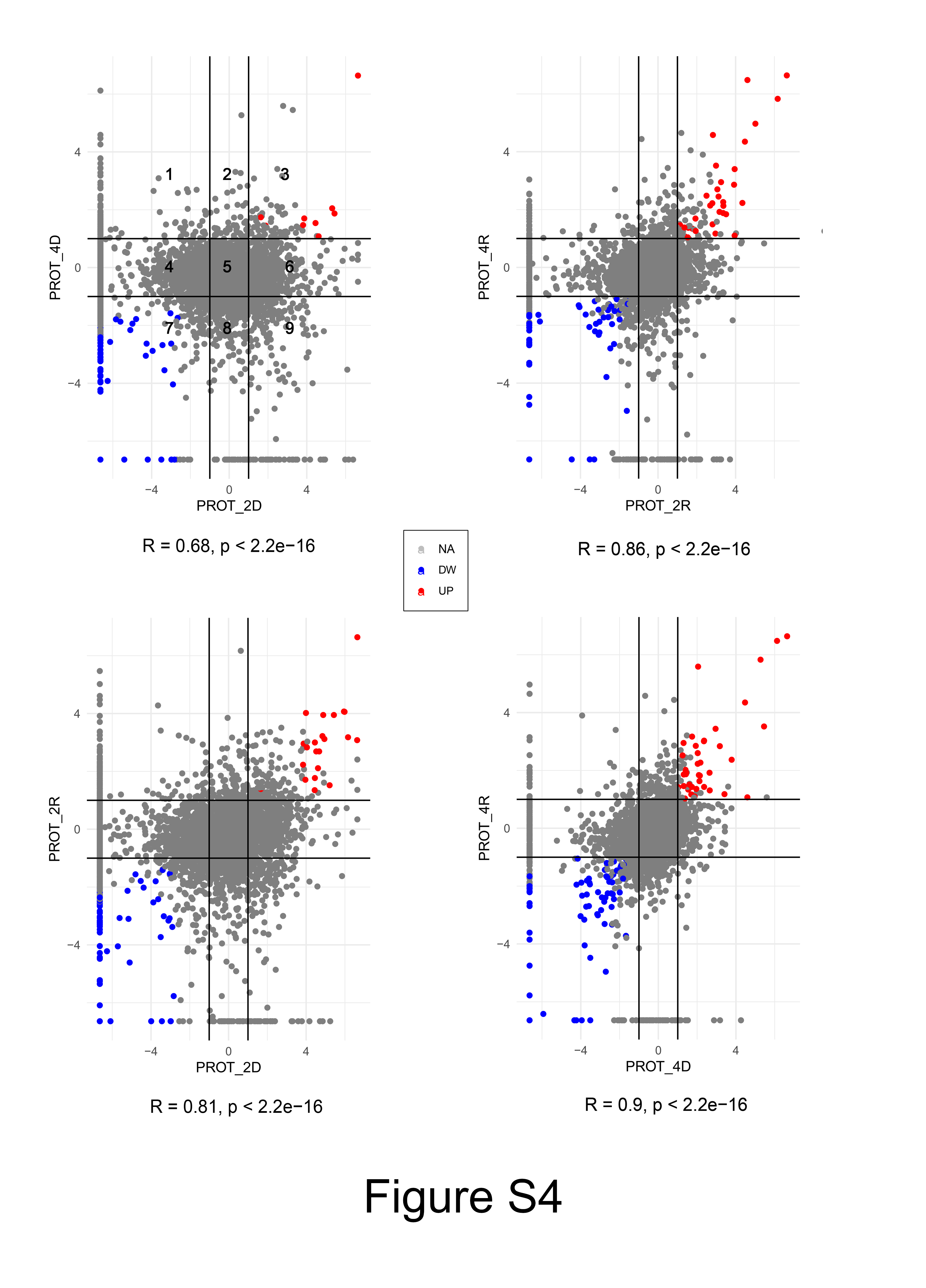

Supplement: Supplementary file 15 — Supplementary file15 (TIF 1722 KB) [file 248_2023_2213_MOESM15_ESM.tif]

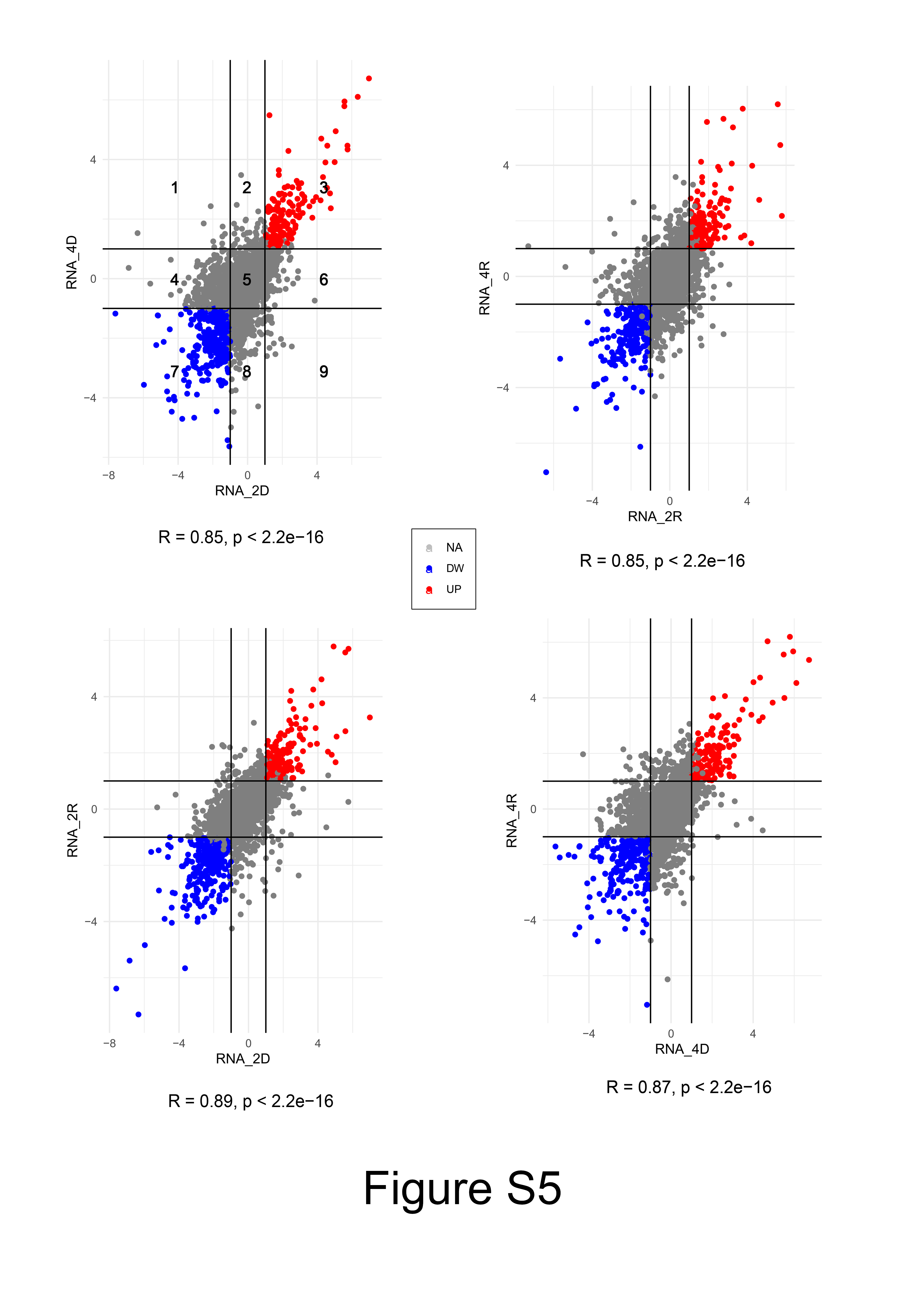

Supplement: Supplementary file 16 — Supplementary file16 (TIF 1463 KB) [file 248_2023_2213_MOESM16_ESM.tif]
