# Supplementary material for: Comparative Transcriptomic and Proteomic Analyses Provide New Insights into the Tolerance to Cyclic Dehydration in a Lichen Phycobiont
Source: Microb Ecol. 2023 Apr 11;86(3):1725–39. doi: 10.1007/s00248-023-02213-x (PMC10497648; doi:10.1007/s00248-023-02213-x)
Supplement: Supplementary file 9 — Supplementary file9 (DOCX 6 KB) [file 248_2023_2213_MOESM9_ESM.docx]

**Table S2:** Assembly quality metrics. Quality metrics of transcripts.

| **Sample** | **Total Number** | **Total Length** | **Mean Length** | **N50** | **N70** | **N90** | **GC(%)** |
| --- | --- | --- | --- | --- | --- | --- | --- |
| **C1** | 36188 | 75247331 | 2079 | 3511 | 2505 | 1331 | 54.85 |
| **C2** | 31373 | 60587471 | 1931 | 3284 | 2378 | 1235 | 54.81 |
| **C3** | 63401 | 117290901 | 1849 | 3533 | 2502 | 1124 | 54.91 |
| **2D1** | 26127 | 52237795 | 1999 | 3399 | 2451 | 1283 | 55.26 |
| **2D2** | 25023 | 48033720 | 1919 | 3269 | 2354 | 1236 | 55.09 |
| **2D3** | 44030 | 68353859 | 1552 | 3149 | 2138 | 840 | 55.06 |
| **2R1** | 43832 | 95254239 | 2173 | 3709 | 2669 | 1399 | 55.1 |
| **2R2** | 64086 | 111200497 | 1735 | 3336 | 2324 | 1010 | 54.91 |
| **2R3** | 61774 | 113286080 | 1833 | 3435 | 2406 | 1118 | 54.93 |
| **4D1** | 30617 | 62579676 | 2043 | 3551 | 2521 | 1284 | 55.06 |
| **4D2** | 54489 | 95732906 | 1756 | 3395 | 2377 | 1026 | 54.74 |
| **4D3** | 33138 | 67621664 | 2040 | 3508 | 2499 | 1305 | 54.84 |
| **4R1** | 37671 | 82752520 | 2196 | 3690 | 2679 | 1414 | 54.8 |
| **4R2** | 37152 | 78646842 | 2116 | 3539 | 2554 | 1358 | 54.75 |
| **4R3** | 56799 | 93466758 | 1645 | 3247 | 2240 | 906 | 54.78 |

Samples: replicates for control conditions (C1-C3), replicates for desiccation conditions after four (4D1-4D3) and two (2D1-2D3) D/R cycles, replicates for rehydration conditions after four (4R1-4R3) and two (2R1-2R3) D/R cycles.

Total Number: total number of transcripts.

Total Length: read length of transcripts.

Mean Length: average length of transcripts.
