# Supplementary material for: Comparative Transcriptomic and Proteomic Analyses Provide New Insights into the Tolerance to Cyclic Dehydration in a Lichen Phycobiont
Source: Microb Ecol. 2023 Apr 11;86(3):1725–39. doi: 10.1007/s00248-023-02213-x (PMC10497648; doi:10.1007/s00248-023-02213-x)
Supplement: Supplementary file 10 — Supplementary file10 (DOCX 6 KB) [file 248_2023_2213_MOESM10_ESM.docx]

**Table S3:** Clustering quality metrics. Quality metrics of Unigenes.

| **Sample** | **Total Number** | **Total Length** | **Mean Length** | **N50** | **N70** | **N90** | **GC(%)** |
| --- | --- | --- | --- | --- | --- | --- | --- |
| **C1** | 30390 | 71902417 | 2365 | 3574 | 2579 | 1426 | 54.93 |
| **C2** | 26163 | 57632434 | 2202 | 3334 | 2444 | 1339 | 54.87 |
| **C3** | 51358 | 110875909 | 2158 | 3601 | 2592 | 1278 | 54.95 |
| **2D1** | 21453 | 49369112 | 2301 | 3447 | 2511 | 1379 | 55.32 |
| **2D2** | 20495 | 45605618 | 2225 | 3328 | 2429 | 1351 | 55.16 |
| **2D3** | 34346 | 64125187 | 1867 | 3230 | 2241 | 1038 | 55.11 |
| **2R1** | 36594 | 90992372 | 2486 | 3767 | 2742 | 1501 | 55.15 |
| **2R2** | 51446 | 104614180 | 2033 | 3407 | 2409 | 1159 | 54.94 |
| **2R3** | 50201 | 107273569 | 2136 | 3512 | 2491 | 1258 | 54.96 |
| **4D1** | 25466 | 59531640 | 2337 | 3608 | 2592 | 1387 | 55.13 |
| **4D2** | 44022 | 90451971 | 2054 | 3463 | 2465 | 1183 | 54.75 |
| **4D3** | 27750 | 64561131 | 2326 | 3561 | 2575 | 1409 | 54.89 |
| **4R1** | 31930 | 79350170 | 2485 | 3752 | 2749 | 1509 | 54.86 |
| **4R2** | 31377 | 75140475 | 2394 | 3603 | 2626 | 1451 | 54.82 |
| **4R3** | 45378 | 88278827 | 1945 | 3325 | 2343 | 1086 | 54.82 |
| **All-Unigene** | 98977 | 316703266 | 3199 | 4671 | 3421 | 1984 | 54.69 |

**Samples:** replicates for control conditions (C1-C3), replicates for desiccation conditions after four (4D1-4D3) and two (2D1-2D3) D/R cycles, replicates for rehydration conditions after four (4R1-4R3) and two (2R1-2R3) D/R cycles.

**Total Number:** total number of Unigenes.

**Total Length:** read length of Unigenes.

**Mean Length:** average length of Unigenes.
