# Supplementary material for: Comparative Transcriptomic and Proteomic Analyses Provide New Insights into the Tolerance to Cyclic Dehydration in a Lichen Phycobiont
Source: Microb Ecol. 2023 Apr 11;86(3):1725–39. doi: 10.1007/s00248-023-02213-x (PMC10497648; doi:10.1007/s00248-023-02213-x)
Supplement: Supplementary file 11 — Supplementary file11 (DOCX 7 KB) [file 248_2023_2213_MOESM11_ESM.docx]

**Table S4**  Quality metrics of Unigenes.

| Sample | Total Number | Total Length | Mean Length | N50 | N70 | N90 | GC(%) |
| --- | --- | --- | --- | --- | --- | --- | --- |
| C1_1 | 30,390 | 71,902,417 | 2,365 | 3,574 | 2,579 | 1,426 | 54.93 |
| C2_1 | 26,163 | 57,632,434 | 2,202 | 3,334 | 2,444 | 1,339 | 54.87 |
| C3_1 | 51,358 | 110,875,909 | 2,158 | 3,601 | 2,592 | 1,278 | 54.95 |
| D1_1 | 25,466 | 59,531,640 | 2,337 | 3,608 | 2,592 | 1,387 | 55.13 |
| D1_2 | 21,453 | 49,369,112 | 2,301 | 3,447 | 2,511 | 1,379 | 55.32 |
| D2_1 | 44,022 | 90,451,971 | 2,054 | 3,463 | 2,465 | 1,183 | 54.75 |
| D2_2 | 20,495 | 45,605,618 | 2,225 | 3,328 | 2,429 | 1,351 | 55.16 |
| D3_1 | 27,750 | 64,561,131 | 2,326 | 3,561 | 2,575 | 1,409 | 54.89 |
| D3_2 | 34,346 | 64,125,187 | 1,867 | 3,230 | 2,241 | 1,038 | 55.11 |
| R1_1 | 31,930 | 79,350,170 | 2,485 | 3,752 | 2,749 | 1,509 | 54.86 |
| R1_2 | 36,594 | 90,992,372 | 2,486 | 3,767 | 2,742 | 1,501 | 55.15 |
| R2_1 | 31,377 | 75,140,475 | 2,394 | 3,603 | 2,626 | 1,451 | 54.82 |
| R2_2 | 51,446 | 104,614,180 | 2,033 | 3,407 | 2,409 | 1,159 | 54.94 |
| R3_1 | 45,378 | 88,278,827 | 1,945 | 3,325 | 2,343 | 1,086 | 54.82 |
| R3_2 | 50,201 | 107,273,569 | 2,136 | 3,512 | 2,491 | 1,258 | 54.96 |
| All-Unigene | 98,977 | 316,703,266 | 3,199 | 4,671 | 3,421 | 1,984 | 54.69 |

**Sample:**Sample name

**Total Number:**The total number of Unigenes

**Total Length:**The read length of Unigenes

**Mean Length:**The average length of Unigenes

**N50:** The N50 length is used to determine the assembly continuity, the higher the better.N50 is a weighted median statistic that 50% of the total length is contained in transcripts that are equal to or larger than this value.

**N70:**Similar to N50

**N90:**Similar to N50

**GC(%):**the percentage of G and C bases in all Unigenes
